# Lipidomics of Thalassiosira pseudonana under Phosphorus Stress Reveal Underlying Phospholipid Substitution Dynamics and Novel Diglycosylceramide Substitutes

**DOI:** 10.1128/AEM.02034-17

**Published:** 2018-03-01

**Authors:** Jonathan E. Hunter, Joost Brandsma, Marcus K. Dymond, Grielof Koster, C. Mark Moore, Anthony D. Postle, Rachel A. Mills, George S. Attard

**Affiliations:** a Ocean and Earth Science, University of Southampton, National Oceanography Centre Southampton, Southampton, United Kingdom; b Institute for Life Sciences, University of Southampton, Southampton, United Kingdom; c Faculty of Medicine, University of Southampton, Southampton General Hospital, Southampton, United Kingdom; d Division of Chemistry, School of Pharmacy and Biomolecular Sciences, University of Brighton, Brighton, United Kingdom; e Chemistry, University of Southampton, Southampton, United Kingdom; University of Georgia

**Keywords:** Thalassiosira pseudonana, phospholipid, sphingolipid, diatom, lipidomics, phosphorus, stress, limitation, substitution, biomarker

## Abstract

Phytoplankton replace phosphorus-containing lipids (P-lipids) with non-P analogues, boosting growth in P-limited oceans. In the model diatom Thalassiosira pseudonana, the substitution dynamics of lipid headgroups are well described, but those of the individual lipids, differing in fatty acid composition, are unknown. Moreover, the behavior of lipids outside the common headgroup classes and the relationship between lipid substitution and cellular particulate organic P (POP) have yet to be reported. We investigated these through the mass spectrometric lipidomics of P-replete (P^+^) and P-depleted (P^−^) T. pseudonana cultures. Nonlipidic POP was depleted rapidly by the initiation of P stress, followed by the cessation of P-lipid biosynthesis and per-cell reductions in the P-lipid levels of successive generations. Minor P-lipid degradative breakdown was observed, releasing P for other processes, but most P-lipids remained intact. This may confer an advantage on efficient heterotrophic lipid consumers in P-limited oceans. Glycerophosphatidylcholine (PC), the predominant P-lipid, was similar in composition to its betaine substitute lipid. During substitution, PC was less abundant per cell and was more highly unsaturated in composition. This may reflect underlying biosynthetic processes or the regulation of membrane biophysical properties subject to lipid substitution. Finally, levels of several diglycosylceramide lipids increased as much as 10-fold under P stress. These represent novel substitute lipids and potential biomarkers for the study of P limitation *in situ*, contributing to growing evidence highlighting the importance of sphingolipids in phycology. These findings contribute much to our understanding of P-lipid substitution, a powerful and widespread adaptation to P limitation in the oligotrophic ocean.

**IMPORTANCE** Unicellular organisms replace phosphorus (P)-containing membrane lipids with non-P substitutes when P is scarce, allowing greater growth of populations. Previous research with the model diatom species Thalassiosira pseudonana grouped lipids by polar headgroups in their chemical structures. The significance of the research reported here is threefold. (i) We described the individual lipids within the headgroups during P-lipid substitution, revealing the relationships between lipid headgroups and hinting at the underlying biochemical processes. (ii) We measured total cellular P, placing P-lipid substitution in the context of the broader response to P stress and yielding insight into the implications of substitution in the marine environment. (iii) We identified lipids previously unknown in this system, revealing a new type of non-P substitute lipid, which is potentially useful as a biomarker for the investigation of P limitation in the ocean.

## INTRODUCTION

Diatoms are a diverse group of eukaryotic microalgae responsible for as much as 25% of global and 50% of oceanic annual primary production (1). Lipids, hydrophobic or amphipathic biological molecules (2, 3), make up 25 to 45% of the total dry weight of diatoms (4), representing a major pool of organic carbon. In the equatorial Pacific Ocean, for example, lipids account for 23% of the organic, total planktonic carbon (5).

Lipid biosynthesis involves a network of reactions that, through the exchange of polar headgroups and fatty acids, generate a rich variety of lipids (3, 6). Remodeling of a cell's lipidome (the entirety of its cellular lipids) in response to environmental conditions is common in unicellular organisms (7, 8). One such remodeling mechanism utilizes the replacement of membrane glycerophospholipids with nonphosphorus glycerolipid counterparts when an organism is subjected to phosphorus (P) stress or starvation (7–10). This response allows a phytoplankter to reduce its P demands in P-limited environments (9), such as the Sargasso Sea or the eastern Mediterranean Sea. These oligotrophic regions contain very low bioavailable phosphate (PO_4_^3−^) concentrations (typically <10 nmol liter^−1^) (9, 11, 12). The reduction in total P demand confers a considerable growth advantage by allowing for the prioritization of nonsubstitutable functions, such as nucleic acid synthesis, over phospholipid biosynthesis (9, 13).

The marine diatom Thalassiosira pseudonana has been used as a model organism to study the effects of P starvation on lipid remodeling (8, 9). Under phosphorus-replete (P^+^) growth conditions, T. pseudonana synthesizes glycerophosphatidylcholine (PC), glycerophosphatidylglycerol (PG), and glycerophosphatidylethanolamine (PE) (8, 9). In contrast, when grown under P^−^ conditions, T. pseudonana synthesizes the nitrogen-containing betaine lipid diacylglycerylcarboxyhydroxymethylcholine (DGCC), which is normally undetectable under P^+^ conditions (9). The increase in DGCC levels is concomitant with a decrease in PC levels, and it is thought that the two physicochemically similar zwitterionic lipids can substitute for each other without loss of membrane function (8, 9, 14). Betaine lipids, including DGCC, are highly abundant in the marine environment (9, 15–18). In addition to the shift between PC and DGCC lipids in P-starved T. pseudonana, PG lipids may be exchanged for sulfur-containing sulfoquinovosyldiacylglycerol (SQDG) (8, 9). As such, ratios of the substitute lipid pairs have been considered to be biomarkers of P stress in both marine and freshwater environments (9, 19).

The biosynthetic pathways underlying these processes are well defined for eukaryotic microalgae in general (20–23), and the genome of T. pseudonana has been published (24). The biosynthetic pathway leading to DGCC is conspicuously unknown, beyond the observed incorporation of radiolabeled methionine (25).

PG and the glyceroglycolipids SQDG, monogalactosyldiacylglycerol (MGDG), and digalactosyldiacylglycerol (DGDG) are enriched in the plastid thylakoid membranes (26). Furthermore, these glyceroglycolipids are biosynthesized within the chloroplast via the intermediate diacylglycerol (DAG). In contrast, the biosynthesis of cellular membrane lipids, including PC and PE, is conducted within the endoplasmic reticulum but also proceeds via a DAG intermediate (20–23). As such, the observed total cellular DAG composition may yield insight into lipid metabolism.

Lipid substitution kinetics in T. pseudonana are rapid, resulting in the exchange of the majority of cellular glycerophospholipids with DGCC and SQDG within 48 h of the initiation of P stress (8). P-starved T. pseudonana responds to the resupply of P faster still, restoring the predominance of glycerophospholipids over a 12- to 24-h period (8). P-lipid substitution dynamics have also been examined in other phytoplankton, such as the pennate marine diatom Phaeodactylum tricornutum, resulting primarily in PG-to-SQDG and PC-to-diacylglyceryl hydroxymethyltrimethyl-β-alanine (DGTA) substitution, reflecting a contrasting system (27). In addition, a comprehensive recent study with Emiliania huxleyi illustrates increased DGCC/PC and SQDG/PG ratios, in addition to ultrastructural modifications resulting from P stress (28).

P-lipid substitution in T. pseudonana has, therefore, been well characterized in terms of the total lipid within each of the major polar headgroup classes. However, important unknowns remain. First, the dynamics of the individual lipid chemotypes (differentiated by the fatty acids they bear) subject to P stress remain unknown. Revealing these dynamics has the potential to yield insight into mechanisms of P-lipid substitution, such as the synthesis of DGCC. Second, the effects of P-lipid substitution on particulate organic P (POP) content per cell have not been reported. Consequently, the relationship between the responses associated with lipidic and extralipidic POP remains unknown. Third, previous work has not provided a comprehensive discussion of the relative contributions of two potential underlying mechanisms for P-lipid substitution, i.e., active breakdown of glycerophospholipids and replacement with non-P alternatives versus a simple switch in biosynthesis to the production of non-P lipids (29). Fourth, and finally, the behavior of minor lipid species, outside the predominant headgroup classes, has yet to be studied. The cellular response to P stress is complex and powerful (30). It is expected therefore, that lipids other than the most common and abundant groups—those studied to date—are also affected. These minor species may display novel substitute or biomarker behavior.

We present here the findings of a mass spectrometry-based lipidomics study using both targeted and untargeted methodologies to characterize the lipidic response to P stress in T. pseudonana cultures. These methods, coupled with culture growth monitoring and the determination of dissolved and particulate nutrient concentrations, yielded novel insight into the kinetics of P-lipid substitution in relation to the broader cell response to P stress. In addition, the data on individual lipid species indicated that the declining PC lipid pool became relatively enriched in unsaturated lipid species. Finally, untargeted lipidomic screening revealed diglycosylceramide sphingolipids that displayed lipid substitute/biomarker behavior in P-stressed cultures. These findings offer a deeper understanding of the dynamics of P-lipid substitution in marine phytoplankton, a fundamental mechanism for growth in P-scarce seas, and its cellular implications.

## RESULTS

### Phospholipid substitution and particulate organic phosphorus dynamics.

**FIG 1:**
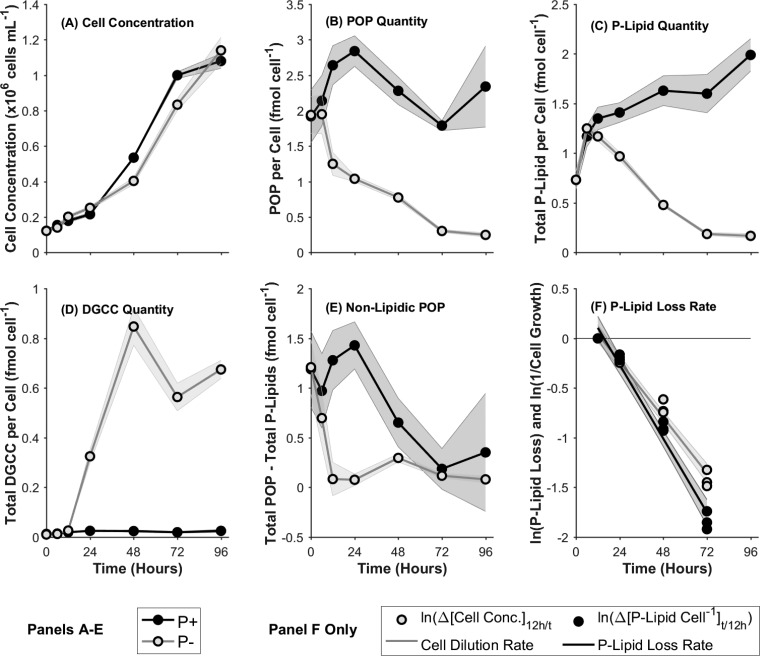
(A to E) Growth curve (expressed as cell concentrations) (A), quantity of particulate organic phosphorus (POP) per cell (B), total phospholipid (P-Lipid) (C), total DGCC (D), and quantity of nonlipidic POP per cell (E) in phosphorus-replete (P^+^) and phosphorus-stressed (P^−^) cultures, with the progression of time. (F) Rate of loss of total phospholipids in comparison with the cell dilution rate. Values are relative to those at 12 h, observed as the time of initiation of P stress, and consequently DGCC biosynthesis and P-lipid substitution/degradation, in P^−^ cultures. Data are means for three biological replicates; shaded regions indicate 1 standard deviation.

During exponential growth (Fig. 1A) between 0 and 72 h, P^+^ cultures grew at a rate of 0.029 ± 0.0013 h^−1^ (doubling time, 23.76 ± 1.08 h). Exponential growth in P^−^ cultures during the same period was marginally slower, at 0.026 ± 0.0016 h^−1^ (*P* < 0.05; doubling time, 27.09 ± 1.78 h). (Statistical significance was determined by a two-tailed, paired equal-variance *t* test of comparisons.) Both P^+^ and P^−^ cultures appeared to be transitioning into stationary phase at 96 h, with maximum populations of 1.08 × 10^6^ ± 4.11 × 10^4^ cells ml^−1^ and 1.14 × 10^6^ ± 7.35 × 10^4^ cells ml^−1^, respectively. Culture viability was high throughout, at >90%, in both P^+^ and P^−^ cultures, except for the P^−^ cultures at 48 h (87.95% ± 1.78%) (see Fig. S1E in the supplemental material); the cultures were therefore considered to be healthy and not subject to stresses other than P up to 72 h.

The dissolved phosphate concentration ([PO_4_^3−^]) (Fig. S1D in the supplemental material) in phosphorus-replete (P^+^) control cultures was in excess (>10 μmol liter^−1^) throughout the 96-h course of the experiment. [PO_4_^3−^] in phosphorus-stressed (P^−^) cultures was 0.80 ± 0.00 μmol liter^−1^ at 0 h and dropped to 0.20 ± 0.00 μmol liter^−1^ after 6 h. This indicates a minor carryover of P from the P-replete seed cultures to the P^−^ culture treatment, which was depleted around 6 to 12 h, subjecting the P^−^ cultures to P stress. The dissolved nitrate concentration ([NO_3_^−^]) and dissolved silicate (orthosilicate ions) concentration ([SiO_4_^4−^]) remained in excess throughout in both P^+^ and P^−^ cultures (Fig. S1A and B in the supplemental material), except for the P^+^ cultures after 72 h, in which [SiO_4_^4−^] was depleted. The cultures were, therefore, not subject to confounding macronutrient stress, with the possible exception of the P^+^ 96-h samples. Taken together, the growth and nutrient data suggest that the 96-h P^+^ samples were entering an Si-limited stationary-growth phase. These data will, therefore, not be considered as a basis for drawing conclusions about P stress below, but they are included for context.

The quantity of particulate organic phosphorus (POP) per cell (Fig. 1B) fell with the progression of P stress over time in the P^−^ cultures. The quantity of POP in P^−^ cultures was initially 1.94 ± 0.26 fmol cell^−1^ and dropped consistently over time to a minimum of 0.25 ± 0.028 fmol cell^−1^ at 96 h. This observed P^−^ minimum was 0.11-fold ± 0.03-fold less (*P* < 0.005) than the P^+^ control samples at 96 h. The POP quantities per cell determined for the P^+^ cultures, between 1.79 ± 0.07 and 2.84 ± 0.22 fmol cell^−1^, are approximately in line with previous observations of 2.8 to 6.2 fmol cell^−1^ for T. pseudonana (31).

Total phospholipid (Fig. 1C) represents the sum of the predominant phospholipid classes: glycerophosphatidylcholine (PC), glycerophosphatidylglycerol (PG), and glycerophosphatidylethanolamine (PE). The individual dynamics of these lipid classes are shown in Fig. S2 in the supplemental material. The quantity of total P-lipid showed an initial increase in both P^+^ and P^−^ cultures between 0 and 6 h, from 0.73 ± 0.08 to 1.25 ± 0.05 fmol cell^−1^ in P^−^ cultures, and declined rapidly thereafter to a minimum of 0.17 ± 0.02 fmol cell^−1^ at 96 h, 0.08-fold ± 0.01-fold less (*P* < 0.005) than the P^+^ control cultures at that time.

The betaine lipid diacylglycerylcarboxyhydroxymethylcholine (DGCC) (Fig. 1D) was absent from P^+^ cultures throughout and from P^−^ cultures before 12 h. Concomitantly with the decline in P-lipid quantities, DGCC quantities increased over time in P^−^ cultures after 12 h to reach a maximum of 0.85 ± 0.075 fmol cell^−1^ at 48 h and 0.67 ± 0.036 fmol cell^−1^ at 96 h.

The quantity of the sulfolipid sulfoquinovosyldiacylglycerol (SQDG) (Fig. S2E in the supplemental material) was 1.31-fold ± 0.14-fold greater (*P* < 0.05) in P^−^ cultures than in P^+^ control cultures at 6 h. SQDG quantities were statistically similar in P^+^ and P^−^ cultures between 12 and 72 h, followed by a 0.71-fold ± 0.045-fold reduction (*P* < 0.005) in P^−^ cultures relative to P^+^ cultures at 96 h. SQDG, therefore, did not change significantly in a consistent manner in response to P stress and did not appear to act as a substitute lipid for PG in this case. This finding contrasts with a previous report on the response of T. pseudonana and other phytoplankton, including cyanobacteria, isolated after longer periods of P starvation (9).

The quantity of diacylglycerol (DAG) (Fig. S2D in the supplemental material) per cell did not differ significantly between P^+^ and P^−^ cultures, except for a 0.58-fold ± 0.10-fold reduction (*P* < 0.05) in P^−^ cultures at 96 h. To our knowledge, DAG has not been characterized in P-stressed T. pseudonana cultures previously and did not differ significantly and consistently in response to P stress.

Neutral storage lipids were not quantified in this study, which was focused on polar and phosphorus-containing membrane lipids. However, for context, it is known that diatoms biosynthesize large reserves of non-P-containing triacylglycerol (TAG) storage lipids subject to P stress (32). This leads to an overall increase in total glycerolipids in the similarly sized pennate diatom P. tricornutum, subject to P stress (27).

### P-lipids versus nonlipidic particulate organic phosphorus.

The difference between the quantities of total POP and total P-lipids (Fig. 1E) was used to investigate the dynamics of non-lipid-associated (nonlipidic) POP within the overarching P stress scenario. In P^−^ cultures, the quantity of nonlipidic POP declined rapidly from 1.21 ± 0.27 to 0.083 ± 0.17 fmol cell^−1^ over the course of the first 12 h, remaining approximately constant thereafter. Nonlipid POP in P^+^ cultures, in comparison, remained statistically constant during the first 12 h, decreasing over the course of 72 h from 1.19 ± 0.38 to 0.19 ± 0.12 fmol cell^−1^.

### Degradation/interconversion versus substitution by *de novo* synthesis.

The rates of change in the P-lipid quantity per cell and in cell concentration were calculated (Fig. 1F) to enable the distinction between a per-cell “dilution,” whereby the original P-lipid was divided up among successive generations of progeny cells, and chemical degradation and breakdown of the original P-lipid. The observations were made relative to the 12-h samples due to the initiation of P-lipid substitution at that time, as evidenced by the depletion of dissolved P (Fig. S1D in the supplemental material) and the commencement of DGCC synthesis (Fig. 1D). The rate of P-lipid loss per cell, −0.031 ± 0.0015 h^−1^, was 32.29% ± 10.91% higher (*P* < 0.005) than the dilution rate by culture growth, −0.023 ± 0.0015 h^−1^ (Fig. 1F). The loss at a rate greater than the rate of cell dilution is indicative of degradative breakdown.

In addition, the difference in total phospholipid per unit volume of culture between a given time (*t*) and 12 h in the P^−^ cultures (ΔPL_*t* − 12 h_) was also used to assess the total quantity of lipid per culture, regardless of per-cell quantities (Fig. S2F in the supplemental material). At 48 and 72 h in P^−^ cultures, ΔPL_*t* − 12 h_ values were −42.27 ± 18.19 pmol ml^−1^ (*P* < 0.05) and −80.47 ± 20.03 pmol ml^−1^ (*P* < 0.005), respectively, indicating a net degradation of the total phospholipids at these times. The amount of P liberated from P-lipids between 12 and 72 h was 7.66 × 10^7^ ± 1.95 × 10^7^ atoms per daughter cell grown during that time, equivalent to 1.06 ± 0.55 diploid genomes per cell (8, 33). Therefore, the P released from P-lipid breakdown appeared sufficient to cover DNA biosynthesis requirements for growth during this time.

The maximum degradative decrease (ΔPL_*t* − 12 h_) at 72 h (Fig. S2F in the supplemental material) corresponded to a loss of 34.10% ± 8.75% of the maximum total phospholipid observed at 12 h in P^−^ cultures. This value is consistent with the P-lipid degradative loss rate as calculated above (32.29% ± 10.91% higher than the dilution rate by culture growth [*P* < 0.005]) over the same interval. In comparison, the change in total DGCC (ΔDGCC_*t* − 12 h_) showed a rapid increase over time, reaching +464.90 ± 50.05 pmol ml^−1^ at 72 h. Taking these findings together, the molar degradative decrease in PL_*t* − 12 h_ was equivalent to 17.31% ± 4.69% of the increase in DGCC.

### Fatty acid level response to phosphorus stress.

**FIG 2:**
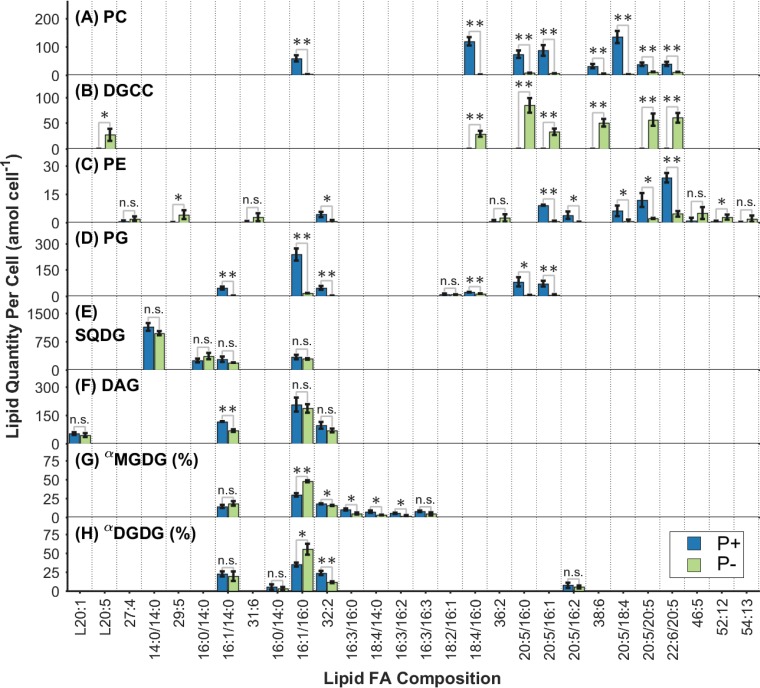
Quantities of individual lipid molecular species per cell within each of the lipid classes measured at 72 h in P^+^ and P^−^ cultures. All quantities are in attomoles per cell, except for MGDG and DGDG. ^α^, relative abundances are expressed as percentages for MGDG (G) and DGDG (H). Molecular species contributing <5% of the total quantity in their lipid headgroup class in both treatments were excluded for the sake of brevity. The predominant fatty acyl (FA) combinations are shown where identified and consistent across the present lipids. Data are means for three biological replicates; error bars, 1 standard deviation. Statistical significance by a two-tailed, paired equal-variance *t* test is indicated by asterisks (*, *P* < 0.05; **, *P* < 0.005); n.s., not significant.

Differences in the quantities of individual lipid species between P^+^ and P^−^ cultures at 72 h are shown in Fig. 2. All PC species (Fig. 2A) showed dramatically lower quantities per cell in P^−^ cultures than in P^+^ control cultures. Some differences in the magnitude of this decrease were evident from the data: PC(18:4/16:0) and PC(20:5/18:4) showed the largest decreases, 0.018-fold ± 0.0035-fold (*P* < 0.005) and 0.023-fold ± 0.037-fold (*P* < 0.005), respectively. PC(16:1/16:0), PC(20:5/16:1), PC(20:5/16:0), and PC(38:6) showed highly significant, intermediate decreases between 0.053-fold ± 0.011-fold and 0.15-fold ± 0.043-fold. In contrast, the highly unsaturated PC(22:6/20:5) and PC(20:5/20:5) species displayed the smallest decreases, 0.28-fold ± 0.054-fold (*P* < 0.005) and 0.30-fold ± 0.052-fold (*P* < 0.005), respectively.

Betaine lipid DGCC species (Fig. 2B) occurred only in P^−^ cultures; levels were below detection in P^+^ control cultures. At 72 h, the five most abundant DGCC species in P^−^ cultures were DGCC(20:5/16:0), DGCC(22:6/20:5), DGCC(20:5/20:5), DGCC(38:6), and DGCC(20:5/16:1), with quantities of 84.28 ± 14.14, 60.08 ± 9.39, 56.56 ± 11.87, 50.56 ± 7.40, and 33.05 ± 6.16 amol cell^−1^, respectively.

The quantities of glycerophospholipid PE species (Fig. 2C) were lower in P^−^ cultures than in P^+^ control cultures. Levels of the predominant PE lipid species PE(22:6/20:5), PE(20:5/20:5), and PE(20:5/16:1) decreased by 0.19-fold ± 0.065-fold (*P* < 0.005), 0.18-fold ± 0.060-fold (*P* < 0.05), and 0.07-fold ± 0.028-fold (*P* < 0.005), respectively, under P stress.

The glycerophospholipid PG (Fig. 2D) behaved in line with PC and generally showed lower quantities per cell for its molecular species in P^−^ cultures than in P^+^ control cultures. The predominant chemotypes, PG(16:1/16:0) (*P* < 0.005), PG(20:5/16:0) (*P* < 0.05), PG(20:5/16:1) (*P* < 0.005), PG(16:1/14:0) (*P* < 0.005), and PG(32:2) (*P* < 0.005), all exhibited decreases between 0.062-fold ± 0.027-fold and 0.11-fold ± 0.048-fold subject to P stress.

Bivariate analyses (see Fig. S3 in the supplemental material) were used to compare the relative abundances of individual lipid species within pairs of lipid classes in order to provide an indication of similarity in their fatty acid distributions. Overall, in considering the distributional similarity between all the lipid classes measured in both treatments, at all time points between 12 and 72 h, two primary clusters were observed. The first cluster, which comprised PC, its substitute DGCC, and PE, showed similar fatty acid distributions, with an average pairwise correlation coefficient (*r*) of 0.68 ± 0.25. In a second cluster, comprising PG, DAG, MGDG, and DGDG, fatty acid distributions were highly cross-correlated (*r* = 0.87 ± 0.10). In addition, these two clusters were highly distinct from each other (*r* = 0.074 ± 0.18). Interestingly, DGCC was moderately correlated with DAG at 12 h (*r* = 0.53 ± 0.023) but showed a weak relationship or none thereafter. DGCC abundance at 12 h was, however, negligible compared to that at 24 h and later.

The absolute quantities of degradative loss of individual PC lipid species were weakly correlated with DGCC gains (see Fig. S4D, E, and F in the supplemental material) for time intervals between 24 and 48 h and between 48 and 72 h in P^−^ cultures, with coefficients of determination (*R*^2^) between 0.3 (*P* < 0.005) and 0.17 (*P* < 0.05), respectively. The linear regression coefficients were consistent with 18 and 20%, respectively, of the DGCC synthesized during these time intervals originating as recycled diglyceride moieties from degraded PC. This estimate is also consistent with the cumulative degradation of PC between 12 and 72 h, presented above, of 17.31% ± 4.69% of the increase in DGCC at the total class level (Fig. S2F in the supplemental material).

### Fatty acid level variability during P-replete and P-depleted growth.

**FIG 3:**
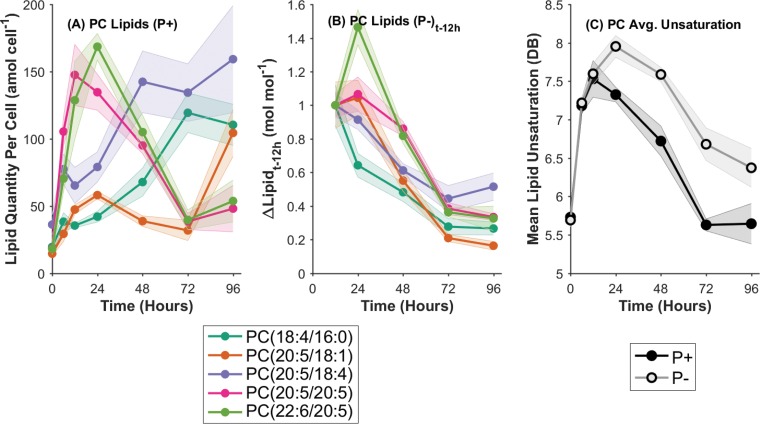
(A) Quantities of the five most abundant individual PC lipid molecular species per cell, over time, in P^+^ control cultures only. (B) Changes in the concentrations of the top five PC lipid molecular species between the initiation of P stress (12 h) and the time indicated (*t*) in P^−^ cultures only. (C) Average unsaturation of the total PC lipid pool in P^+^ and P^−^ treatments with the progression of time. DB, double bonds. Data are means for three biological replicates; shaded regions indicate 1 standard deviation.

In order to study the temporal dynamics of P-lipids in the context of P stress at the individual lipid species level, the five most abundant PC lipids, as the predominant components of the total P-lipid pool, were analyzed in isolation. First, the underlying dynamics of the P^+^ control cultures were considered (Fig. 3A). The top five PC molecular species, ranked by their maximum quantity per cell, appeared to conform to one of two behaviors. The highly unsaturated PC(20:5/20:5) and PC(22:6/20:5) chemotypes displayed a sharp increase between 0 and 12 to 24 h with the initiation of exponential growth, followed by an equally sharp decline in abundance to 72 h. PC(22:6/20:5), for example, rose from 18.11 ± 4.39 amol cell^−1^ at 0 h to 168.57 ± 9.83 amol cell^−1^ at 24 h (*P* < 0.005) before dropping back to 39.67 ± 7.28 amol cell^−1^ by 72 h (*P* < 0.005). In contrast, the moderately unsaturated species PC(18:4/16:0), PC(20:5/18:1), and PC(20:5/18:4) displayed a consistently increasing trend with the progression of time in P^+^ control cultures. PC(20:5/18:4), for example, increased from 36.21 ± 7.44 amol cell^−1^ at 0 h to 159.26 ± 39.81 amol cell^−1^ at 96 h (*P* < 0.05).

To decouple the P-stress-driven dynamics from the growth-driven dynamics, the change in PC lipid quantity over time, relative to the initiation of P stress in P^−^ cultures at 12 h, was determined (Fig. 3B). Overall, all of the top five PC lipids exhibited a continuous decreasing trend in quantity between 12 and 96 h subject to P stress. The magnitude of change differed between different lipid species; PC(20:5/18:4) and PC(20:5/18:1) decreased by 0.52-fold ± 0.081-fold and 0.16-fold ± 0.025-fold, respectively (*P*, <0.005 for both). The response of PC(22:6/20:5) was an exception, showing an initial 1.46-fold ± 0.11-fold increase between 12 and 24 h, followed by a large 0.33-fold ± 0.073-fold decrease (relative to 12 h) (*P* < 0.005). The least unsaturated of the major PC lipids, PC(18:4/16:0) showed the most rapid initial decrease between 12 and 24 h, dropping by 0.64-fold ± 0.070-fold.

The variability in the responses of these PC lipids to P stress was reflected in the changing average unsaturation of the PC lipid pool over time (Fig. 3C). Average PC unsaturation (the total number of fatty acid double bonds) in both P^+^ control and P^−^ cultures displayed sharp increases between 0 and 12 h, from 5.73 ± 0.098 to 7.53 ± 0.24 double bonds in P^+^ cultures. After 12 h, the results diverged for the two treatments, concomitantly with the initiation of P stress. The P^+^ cultures then displayed a decrease in average PC unsaturation to 5.63 ± 0.077 double bonds at 72 h, remaining constant thereafter. The P^−^ cultures, in contrast, continued increasing to a maximum of 7.95 ± 0.14 double bonds at 24 h, followed by a decline until 96 h to 6.37 ± 0.25 double bonds, but remained more highly unsaturated than the P^+^ control cultures throughout.

These observations co-occur with the decrease in per-cell quantities of P-lipids, and the minor degree of degradative breakdown, in P^−^ cultures, described above (Fig. 1C). Therefore, the initial shift to higher unsaturation in P^−^ cultures indicates that P stress causes either a preferential degradation of less-saturated PC lipids or the continued synthesis of small quantities of highly unsaturated PC lipids during early P stress between 12 and 24 h (both mechanisms appear to be supported by Fig. 3B). At later time points, this process ceases, and the average PC unsaturation begins to decrease and trend toward (but not achieve) convergence with the P^+^ control values. Overall, a more highly unsaturated PC lipid pool occurs subject to P stress (Fig. 3C).

### Untargeted lipidomic screening.

**FIG 4:**
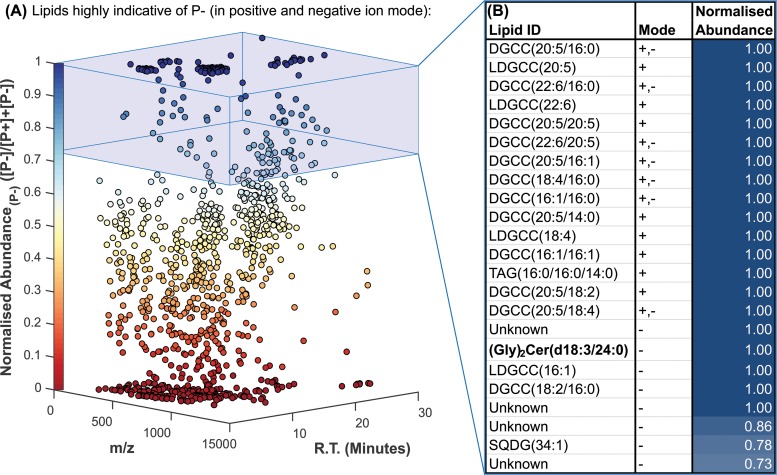
Untargeted lipidomic screening determined a total of 1,161 different molecular ions in the 72-h P^+^ and P^−^ cultures by use of positive- and negative-ion mass spectrometry. (A) The normalized abundance in P^−^ cultures, indicating how strongly a given ion was associated with the P-stressed cultures, is plotted against the mass-to-charge ratio (*m/z*) and chromatographic retention time (R.T.). (B) The top 15 ions associated with the P-stressed cultures in the positive and negative modes are identified, where possible, by database matching and MS^2^ fragmentation analysis.

Lipid species were ranked according to their normalized abundance, in descending order from the most strongly increased under P stress (Fig. 4B). The betaine lipid DGCC dominated the positive-ion results, comprising 14 of the top 15 ions. DGCC species containing eicosapentaenoic (20:5), docosahexaenoic (22:6), and palmitic (16:0) fatty acids displayed the greatest differential increases and were hence the highest ranked.

Most remarkably, a 1,014.7112-Da species, corresponding to a diglycosylceramide with a dihydroxy(18:3) long-chain base and a 24:0 fatty amide [(Gly)_2_Cer(d18:3/24:0)] with a formic acid adduct, displayed an absence-presence response to P stress. The chemical structure, and its determination by tandem mass spectrometry (MS^2^) fragmentation in both the negative- and positive-ion modes, is displayed in Fig. S5 in the supplemental material.

**FIG 5:**
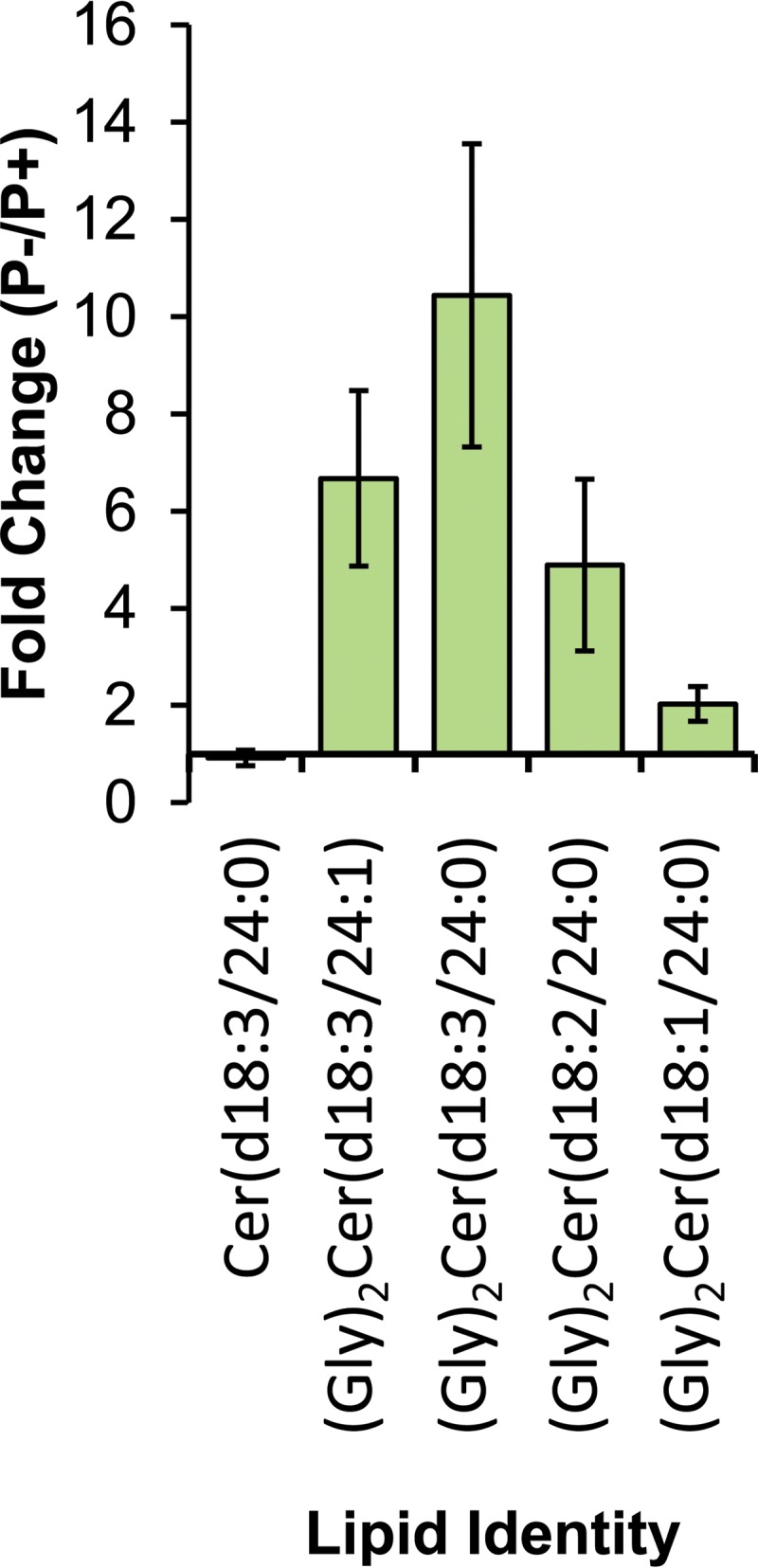
Fold change, subject to P stress, in observed ions relative to (Gly)_2_Cer(d18:3/24:0), in positive-ion mode. Long-chain base and fatty acid amide fragments were observed in support of each of the assignments in positive MS^2^. Data represent means for biological triplicate samples; error bars, 1 standard deviation. Assignments represent the primary fatty acyl configurations, as determined by the abundances of the fatty acyl fragments in the MS^2^ spectra.

Several lipids closely related to (Gly)_2_Cer(d18:3/24:0) were subsequently observed by use of more-sensitive semitargeted analyses in the positive-ion mode (Fig. 5). Of these, (Gly)_2_Cer(d18:3/24:0) itself responded most strongly to P stress, with a 10.43-fold ± 3.12-fold increase in the P^−^ culture over the level in the P^+^ culture (*P* < 0.005). All the glycosphingolipids displayed statistically significant increases subject to P stress, albeit of various magnitudes. (Gly)_2_Cer(d18:3/24:1), (Gly)_2_Cer(d18:2/24:0), and (Gly)_2_Cer(d18:1/24:0) levels increased by 6.67-fold ± 1.81-fold, 4.89-fold ± 1.76-fold, and 2.03-fold ± 0.36-fold, respectively, in P^−^ cultures over levels in P^+^ cultures (*P*, <0.005 for all three lipids). The ceramide equivalent lipid Cer(d18:3/24:0), without the diglycosyl headgroup, was also detected. In contrast, its differential abundance between P^−^ and P^+^ cultures showed no significant change.

## DISCUSSION

### Phospholipid substitution and particulate organic phosphorus dynamics.

We find that the dynamics of P-lipid substitution in T. pseudonana follow primarily a cessation of P-lipid net biosynthesis with a minor degree of P-lipid net breakdown (Fig. 1). The amount of P liberated by degradative breakdown of P-lipids was enough to synthesize approximately 1 diploid genome per cell during the same period of P-stressed growth, one-fourth of previous estimates (8). The majority of the original cellular P-lipids were retained and diluted in per-cell terms with the progression of culture growth by cell division, a finding consistent with the observed decrease in per-cell POP levels. No appreciable growth limitation occurred on these time scales, reflecting the great capacity of this organism, not least with respect to its lipidomic flexibility, to adapt to low P environments (30).

The quantity of nonlipidic POP per cell was observed to decrease to near zero after 12 h. This finding warrants discussion, given the expected obligate cellular P requirements for essential genomic and other biomolecules. The minimum observed value for nonlipidic POP, 0.076 ± 0.058 fmol cell^−1^ at 24 h, is just barely sufficient to account for the estimated P associated with DNA per cell (63.74% ± 48.64% [estimated from reference 33]) when the upper standard deviation limit is included. Therefore, it is possible that the total POP quantities measured represent a slight underestimate. This is supported by the POP per-cell quantities measured in P^+^ cultures, which are shown in Results to be consistent with the lower limits of literature values. Nonetheless, the relative shift from the initial state toward a minimum value after 12 h remains a robust observation appropriate for further discussion.

The quantity of nonlipidic POP declined toward its minimum by 12 h, prior to the initiation of the P-lipid response. The temporal delay in the lipid remodeling response to P stress may be attributable to the prior utilization of P from other intracellular pools. Polyphosphates (polyP) could be one such pool (34); however, these observations conflict with recent environmental observations that suggest polyP enrichment of phytoplankton in P-limited waters (35). To our knowledge, polyP has not been quantified in T. pseudonana to date, so this contradiction cannot be reconciled at this point.

The majority of the original lipid-bound P remained as such and did not appear to be made available for other cellular processes, such as nucleic acid synthesis (8, 27). This does not diminish the ability of the organism to grow subject to reduced P availability by utilizing non-phosphorus-containing lipids rather than phospholipids. The distinction has subtle conceivable ecological implications. Heterotrophic bacteria that consume lipids efficiently (36), for example, may benefit from access to lipidic P content in oligotrophic waters. Additionally, assuming that the use of non-phosphorus-containing lipids rather than phospholipids has little physiological influence on cell growth, the phospholipid content of P-replete cells arguably represents an enigma if this P pool is not strongly repurposed upon the development of P stress.

The stable abundance of SQDG throughout could be interpreted as an adaptation to economize on P requirements in the oceanic environment, in which P is regularly scarce. PG and SQDG, the substitute lipid pair, share at least a partial functional similarity/redundancy in their roles in the thylakoid membranes (10, 26). However, the efficiency of photosystem II is demonstrably compromised in SQDG-deficient algal mutants (37), suggesting that SQDG plays an essential functional role and is not only a non-P substitute for PG.

Overall, this deeper understanding of a powerful phytoplankton adaptation to P stress gives greater context to the resulting dramatic shifts in elemental ratios and phytoplankton growth dynamics, with subsequent implications for both trophic transfer and export flux.

### Fatty acid level response to phosphorus stress.

The quantities of individual PC lipids per cell were highly dynamic in P^+^ control cultures with the progression of time (Fig. 3A). Levels of the most highly unsaturated PC lipids increased markedly in early-exponential growth before declining back to initial levels later, as cultures progressed toward the stationary-growth phase. More-saturated major PC lipids displayed progressively increasing quantities per cell over time. These observations are in agreement with previous reports on the relationship between the unsaturation of eukaryotic phytoplankton glycerolipids and the growth phase (38). The change in the lipid unsaturation state arises from the partitioning between the biosynthesis of polar lipids and that of neutral storage lipids, which proceed via distinct elongation and desaturation pathways (38). During rapid growth, polyunsaturated fatty acids characteristic of the structural polar lipids are biosynthesized. However, during the transition to stationary growth, more-saturated fatty acids are synthesized for incorporation into TAG storage lipids. This is reflected, in part, in the polar glycerolipids due to a degree of cross talk (38).

The variation in membrane glycerolipid composition discussed should have functional implications for membrane biophysics and structure, unless it is homeostatically compensated for in some other manner (39). The complexity and degree of the temporal changes in individual lipid species composition, within P^+^ control cultures, highlight the lipidomic plasticity of T. pseudonana and its highly dynamic nature, both often poorly accounted for in biomarker studies. This behavior contributes to a lack of diagnostic power for chemotaxonomic distinction of phytoplankton that has been reported elsewhere with respect to fatty acids (40) and intact polar lipids (18).

In P^−^ cultures, we observed a preferential degradation of more-saturated PC lipid species and/or a continuing synthesis of highly unsaturated PC lipids, leading to a more highly unsaturated, less-abundant PC lipid pool (constituting the majority of the P-lipids) under P stress (Fig. 3B and C). It could be speculated that this increase in unsaturation represents a homeostatic control on membrane fluidity (as in reference 39) in light of the increasing substitution of DGCC for P-lipids, which may result in differing biophysical characteristics.

The fatty acid composition of DGCC, which increased in cellular abundance with the progression of P stress, was correlated with that of PC (Fig. 2; also Fig. S3 in the supplemental material). This similarity in composition is consistent with the role of DGCC as a substitute lipid for PC in T. pseudonana (8, 9), and the observation provides further evidence for the substitutive link between the two.

The molar quantity of P-lipid breakdown accounted for a minor proportion of the biosynthesized DGCC at the total-class level. In addition, the degradative loss of individual PC lipid species was weakly correlated with the synthesis of their DGCC counterparts (Fig. S4 in the supplemental material). These data are consistent with, but not definitive evidence of, the recycling of diglyceride moieties from phospholipid breakdown for incorporation into newly biosynthesized DGCC. This observation must be further confirmed by an isotopic labeling experiment to unambiguously trace the transformation.

DAG is a known direct precursor of glycerophospholipids (PC, PE) in eukaryotes (20). Under P^−^ conditions, an uncoupling of DAG composition with the glycerophospholipids could be expected if they are not being synthesized. However, DAG composition did not differ between P^+^ and P^−^ conditions, as shown above. Therefore, we would expect the composition of lipid species in DAG to reflect that of PC, the most abundant of its biosynthetic products, under P^+^ conditions, but that was not the case. Furthermore, no consistent correlation was observed between DGCC and DAG. As the precursor to PC biosynthesis (20) and a substructure common to both PC and DGCC, DAG is a potential intermediate in the synthesis of DGCC. This could occur via *de novo* synthesis or could represent a recycling of any diglyceride from PC breakdown via phospholipase C. Again, our observations do not support these hypotheses. PC is synthesized from DAG under P^+^ conditions, yet we cannot observe this via the correlation of their individual lipid species. Based on these results, a role of DAG in the biosynthesis of DGCC cannot be ruled out. Unfortunately, comparison of the various polar lipid distributions did not yield significant insight into the as-yet-unknown biosynthetic pathway leading to DGCC. To our knowledge, nothing appears to be known other than the reported incorporation of ^14^C-labeled methionine into its headgroup in labeling experiments (25, 41). This remains a very conspicuous gap in our understanding of a lipid class that forms a major component of the planktonic lipid pool (42).

Thus, it appears that the DAG observed is utilized primarily by monogalactosyldiacylglycerol synthase as a biosynthetic precursor to MGDG and DGDG (21, 24). These observations lead to the hypothesis that there are two (or more) separate pools of DAG. The first is the larger and/or more slowly turned over DAG pool indicative of MGDG/DGDG synthesis, observed by characterization of the total lipid extract and localized within the chloroplast. The second is the smaller and/or more rapidly turned over DAG pool indicative of PC/PE synthesis, which is not observed. Further investigation of DAG lipid dynamics within isolated subcellular locales may yield further insight into the link between DAG and the biosynthesis of PC and DGCC.

### Untargeted lipidomic screening.

Untargeted lipidomic screening revealed (Gly)_2_Cer(d18:4/24:0), which was approximately 10-fold more abundant in P^−^ than P^+^ cultures. This discovery led to the identification of a series of related molecules differing subtly in the degrees of unsaturation of the long-chain base or the fatty acid amide (Fig. 5). Levels of all of the diglycosylceramide species increased under P stress, differing in the magnitude of change between approximately 2-fold and 10-fold. A Cer(d18:3/24:0) species was also identified and demonstrated contrasting behavior. This putative precursor did not differ in cellular abundance subject to P stress. Chemically identical and related lipids have been reported previously in another marine diatom, Skeletonema costatum (43), and genes encoding sphingolipid biochemistry are also annotated in the genome of T. pseudonana (24). This lends further credence to our identification and highlights these molecules as an area of interest for future research into diatom lipid biochemistry. Glycosylceramides have been assigned several physiological functions, including membrane stability, membrane permeability, and pathogenesis (44). In this context, it appears that they may be acting as nonphosphorus substitute lipids, akin to DGCC (8–10), for another phosphorus-containing lipid and that this drives their differential increase under P stress.

Chemically closely related glycosphingolipids have been identified in virally infected Emiliania huxleyi and have been used as biomarkers for viral infection in the marine environment (45–48). In the same manner, we propose the diglycosylceramides that increase in cellular abundance subject to P stress as candidate biomarkers for the P stress of marine diatoms in the environment. As such, the abundances of these potentially diagnostic biomarkers could be quantified in diatoms collected from the environment of interest. These data could yield additional insight into the level of P stress experienced by the phytoplankton directly, which would be information complementary to the more-routine measurement of dissolved or particulate P concentrations in the medium. Targeted quantification of these lipids would be readily achievable using commercially available lactosylceramide standards (as in reference 43). Such a lipid biomarker could be useful for monitoring P-related biogeochemical processes in the environment, pending further validation. More broadly, the discovery of this sphingolipid behavior, taken together with the E. huxleyi host-virus system, strongly supports phytoplankton sphingolipid biochemistry as an important avenue for future research.

### Conclusions.

We have presented here the comprehensive lipid analysis of phospholipid substitution induced by P stress in the model marine diatom T. pseudonana at the level of individual lipid species, those differing in fatty acid composition, and in tandem with the quantification of cellular particulate organic phosphorus (POP). These data indicate the depletion of a nonlipidic POP fraction prior to the lipid response. This was followed immediately by *de novo* biosynthesis of the substitute lipid DGCC. The majority of the original P-lipid remained intact and was diluted among progeny cells, but a quantity of P equivalent to approximately 1 diploid genome per daughter cell was liberated by degradative breakdown of P-lipids. The distinction between P associated ultimately with lipids, as opposed to genomic material, may have implications for subsequent trophic transfer and export flux.

At the individual lipid level, all major P-lipids were reduced in cellular quantity but in a nonuniform manner. P stress resulted in an increased average degree of unsaturation within the declining PC lipid pool, which represented the predominant P-lipids. These observations indicate a preferential degradation of more-saturated PCs and/or a continuing synthesis of highly unsaturated PC lipids, perhaps in order to regulate membrane fluidity in the context of the major change in membrane composition through substitution. Further, the data were tentatively consistent with the recycling of diglyceride substructures from P-lipid degradation into newly synthesized DGCC.

DAG fatty acyl composition primarily reflected that of the chloroplast-associated glyceroglycolipids and did not yield insight into the biosynthetic pathway of the substitute lipid DGCC. This pathway remains a significant gap in our understanding of a biochemical highly abundant in the marine environment.

Finally, untargeted lipidomic screening revealed a group of diglycosylceramide lipids whose levels increased subject to P stress as much as 10-fold and which constitute nonphosphorus substitute lipids and candidate biomarkers for P stress. Taken together, these findings contribute a new level of understanding of P-lipid substitution in marine phytoplankton, a powerful and widespread mechanism of adaptation to low-P environments with important consequences for macronutrient biogeochemistry and oligotrophic primary production.

## MATERIALS AND METHODS

### Culturing.

Axenic T. pseudonana (1085/12; also designated CCMP1335/3H) was obtained from the Culture Collection of Algae and Protozoa, Scottish Association for Marine Science, United Kingdom. Culture manipulations were performed under sterile conditions in a laminar flow environment.

F/2+Si growth medium (49), based on artificial seawater, was prepared (50) from analytical- or biological-grade components (Fisher Scientific). Seed culture (175 ml) was grown to a mid-log-phase concentration of 1.13 × 10^6^ cells ml^−1^ over 4 days of incubation at 18°C (light/dark cycle, 12 h/12 h; illumination, 123 μmol quanta m^−2^ s^−1^; gentle orbital agitation at 70 rpm).

The seed culture was split (2 parts, 79.6 ml each), and cells were isolated from the medium by filtration (Millipore Steritop; pore size, 0.22 μm). Cells were washed on the filter with 50 ml of P^+^ or P^−^ medium, depending on the treatment, resuspended, and split to form 3 parts of 300 ml for each P^+^ and P^−^ culture. These experimental cultures were incubated as described above and were sampled after 0, 6, 12, 24, 48, 72, and 96 h for size distribution/cell count, viability, dissolved/particulate macronutrients, and lipid extracts.

An aliquot (950 μl) of culture was mixed with a freshly prepared paraformaldehyde solution (170 μl; 34% [wt/vol] distilled water [dH_2_O]) and was stored at 4°C for <24 h before analysis. Cell size distributions were generated with a Beckman Coulter Multisizer 3 Coulter counter. A 70-μm aperture and 3% NaCl electrolyte were used, and the samples were diluted to ensure <10% aperture coincidence concentration. The Coulter counter was calibrated with 5.023-μm polystyrene latex standard beads prior to use (Beckmann Coulter via Meritics Ltd., Dunstable, United Kingdom). Size distributions were used to generate cell concentration values, between particle diameter limits of 3 and 9 μm.

The experimental culture (50 μl) was incubated with Sytox Green dye (Invitrogen Life Technologies, Paisley, United Kingdom) at a concentration of 0.5 μM for 5 min in the dark. An 18-μl volume of this solution was then imaged with a Cellometer Vision Duo system (X100-F101 optics; SD100 slides; Nexcelcom Bioscience via Peqlab, Sarisbury Green, United Kingdom). All cells were counted manually in the bright-field mode and stained, nonviable cells were counted in the fluorescence mode (excitation/emission wavelengths, 470/535 nm).

### Nutrient quantification.

Experimental culture (10 ml) was syringe filtered over precombusted (450°C, 12 h) GF/F filters. The filtrate was stored at −20°C. Filters were dried at 60°C for 24 h and were stored in a desiccator. Particulate phosphorus was determined by following an oxidation procedure described in reference 51, and samples were centrifuged (1,000 × *g*, 10 min, 18°C) before analysis to remove particulates. Nutrient samples were diluted 1/60 (dissolved nutrients [filtrate]) and 3/20 (oxidized particulate nutrients) in Milli-Q dH_2_O and were characterized by segmented flow autoanalysis on an AutoAnalyzer 3 system (Seal Analytical, Fareham, United Kingdom) for phosphorus, nitrate/nitrite, and silicon. POP quantities were corrected for carryover of inorganic phosphate from the growth medium based on the difference between the P^+^ and P^−^ POP quantities measured at 0 h, normalized to the [PO_4_]^3−^ concentration in the P^+^ growth medium for a given sample (as measured by the dissolved phosphate [DIP] assay): $[\left({{\text{POP}}^{\text{P+}}}_{\text{t}0}\right)-({{\text{POP}}^{\text{P}-}}_{\text{t}0})]\times \left[\left({{\text{DIP}}^{\text{P+}}}_{\text{t}})/({{\text{DIP}}^{\text{P+}}}_{\text{t}0}\right)\right]$.

### Lipid extraction.

Lipid samples (20 ml) from the experimental cultures were isolated by syringe filtration as described above. The filtrate was discarded and the filters stored at −78°C until extraction. Total-lipid extracts were prepared using a Bligh-Dyer extraction procedure (52) as modified in reference 53. Solvents were of liquid chromatography (LC)-mass spectrometry (MS) grade (Fisher Scientific). Lipid internal standards were added at quantities adjusted per sample to maintain constant standard/cell equivalent ratios as follows: PC(12:0/12:0), 56.2 amol cell^−1^; PG(12:0/12:0), 77.0 amol cell^−1^; PE(12:0/12:0), 47.6 amol cell^−1^; DAG(20:4/18:0), 99.7 amol cell^−1^; SQDG (mixed extract), 1.27 fmol cell^−1^. Phospholipid/DAG standards were acquired from Avanti Polar Lipids (Alabaster, AL, USA), and SQDG spinach leaf extract was provided by Lipid Products (Surrey, United Kingdom).

A variable volume of the lipid-containing phase was isolated per sample, ensuring that a constant quantity of lipid (including standards), as estimated by cell concentration in a given sample, was infused into the mass spectrometer during analysis (hence accounting for ion suppression and enabling external-standard quantification of DGCC). The optimal lipid quantity for analysis as outlined below was equivalent to the quantity derived from 0.8 × 10^6^ cells (including standards as described above). This fraction was dried under N_2_ and was stored at −20°C until analysis.

### Direct infusion electrospray ionization (ESI)–MS^2^ quantitative analysis.

Mass spectrometric analysis was performed on a Waters Micromass Quattro Ultima triple-quadrupole instrument. Dried samples were dissolved in 250 μl 66% methanol–30% dichloromethane–4% ammonium acetate (300 mM in H_2_O). The sample solution was directly infused into the instrument at 6 μl min^−1^ and was analyzed by tandem MS (MS^2^) analysis of each lipid class (53). In addition, a neutral loss scan of 35 Da, between 350 and 750 Da at a collision energy of 15 eV, was used for the detection of ammoniated DAG molecular ions.

Spectra were processed by despiking, baseline subtraction, isotopic correction, and assignment by a Visual Basic macro (54). The spectrum of the SQDG standard in isolation was taken in triplicate and was used to perform a subtraction for overlapping standard-derived peaks (due to the natural-extract nature of the SQDG standard) based on the dominant peak at 834 Da (SQDG with 34 fatty acyl carbons and 3 double bonds, not detectable in T. pseudonana under P^+^ or P^−^ conditions). DGCC extract (purified by preparative high-performance liquid chromatography [HPLC] from T. pseudonana under P starvation) was provided by Benjamin A. S. Van Mooy (Woods Hole Oceanographic Institute, USA). External-standard calibrations were generated from the addition of DGCC extract (0.094, 0.19, 0.38, 0.75, and 1.5 nmol) to P-replete-culture-grown (hence with no intrinsic DGCC) T. pseudonana total-lipid extracts prepared at optimal, constant cellular lipid/standard concentrations as discussed above. Total DGCC counts were normalized to the chemically similar PC internal standard for the quantification of experimental samples according to the following linear calibration equation: DGCC counts/PC counts = 18.594 × DGCC quantity (*R*^2^ = 0.992).

### Untargeted UPLC-ESI-AutoMS^2^ analysis.

Ultraperformance LC (UPLC)-ESI-AutoMS^2^ analysis was performed on a Dionex UltiMate 3000 UPLC system coupled to a Bruker maXis 3G quadrupole–time of flight (Q-ToF) mass spectrometer with an electrospray ionization source. Lipid samples were dissolved in methanol (200 μl) prior to analysis. A 20-μl injection was taken by autosampler from vials in a cooled sample tray at 5°C. The sample was then chromatographically separated over 30 min with a Waters Acquity UPLC BEH C8 column (2.1 by 100 mm; particle size, 1.7 μm). A constant flow rate of 0.3 ml min^−1^ was used, resulting in back pressures between 26,000 and 46,000 kPa.

Eluent A was water with 0.2% formic acid and 1% 1 M ammonium acetate, and eluent B was methanol with 0.2% formic acid and 1% 1 M ammonium acetate. The column was heated to 50°C, and the postcolumn eluent was cooled to 21°C, throughout the analysis. The following multistep linear gradient was applied, with a constant flow rate of 0.3 ml min^−1^: 35% eluent B at 0 min, increasing to 80% eluent B at 2 min, increasing to 95% eluent B at 12 min, and holding for a further 18 min until the end of the run. Eluent B was decreased to 35% over 0.5 min after the run, and the column was allowed to equilibrate for 4.5 min prior to the next run.

The mass spectrometer was calibrated by direct infusion of a sodium formate solution prior to use (10 mM sodium hydroxide plus 0.2% formic acid in isopropanol-water [1:1]). The observed mass accuracy was 0.4 ppm, determined from the standard deviation from the quadratic calibration curve for calibrant ions up to 1,000 Da, in positive-ion mode. The Q-ToF mass spectrometer yielded a mass resolving power of 21,463.70, determined from full width at half maximum (FWHM) of the internal-standard peak, dilauroylphosphatidylcholine, at 622.4470 Da [M+H]^+^ in positive-ion mode. Full-scan MS was acquired in the positive and negative modes between 30 and 1,500 Da.

During each run (the positive- and negative-ion modes require separate analytical runs), ions above the noise level threshold were subjected to data-dependent MS^2^ fragmentation. The thresholds were set at 2,000 and 1,000 counts in the positive- and negative-ion modes, respectively. Ions with a mass between 500 and 1,500 Da or between 300 and 1,500 Da were subjected to MS^2^ fragmentation in the positive- and negative-ion modes, respectively. The two most abundant precursor ions eluting during an MS scan were fragmented, and after two MS^2^ spectra were acquired for a given ion, they were actively excluded from further MS^2^ for 1 min. Fragmentation for MS^2^ was achieved by collision-induced dissociation (CID) by impact with argon gas, with stepped collision energies for precursor ions of increasing mass. Ions of 300 to 500, 500 to 800, and >800 Da were fragmented with collision energies of 25, 40, and 50 eV, respectively, in positive-ion mode and 25, 30, and 40 eV in negative-ion mode.

### Untargeted data processing.

Bruker CompassXport was used to export the raw data prior to processing with the MZMine 2 software package (55). MZMine was used to generate extracted ion chromatograms and to match these chromatograms between different samples. Integrated peak areas were normalized to the total number of cells extracted and were adjusted for recovery of the internal standard (dilauroylglycerophosphatidylcholine). Peak assignments were based on matching to an extensive, accurate mass, structure query language (SQL) lipid database generated in-house. The database was populated by permutations of fatty acids (chain length/degree of unsaturation) and common glycerolipids/sphingolipids. The complete LIPID MAPS (version 20130306) structural database (56) and MaConDa mass spectrometry contaminants database (57) were also included.

The chemical formulae of database entries were then used to calculate accurate mass *m/z* values based on a list of common molecular ion adducts in ESI-MS (58). Tentative assignments were made by matching precursor mass ions with the theoretical database to within a mass difference of <10 ppm. Database assignments were then confirmed by the identification of supporting MS^2^ fragments in each case. Fragments were assigned within a tolerance of <20 ppm unless otherwise stated.

The MS^2^ data were assigned using an in-house Visual Basic macro, matching fragment ions to a database of common and diagnostic fragments and dynamically generated neutral losses. Matches were made based upon a tolerance of <20 ppm (unless otherwise specified).

## Supplementary Material

Supplemental material
