# Supplementary material for: Lipidomics of Thalassiosira pseudonana under Phosphorus Stress Reveal Underlying Phospholipid Substitution Dynamics and Novel Diglycosylceramide Substitutes
Source: Appl Environ Microbiol. 2018 Mar 1;84(6):e02034-17. doi: 10.1128/AEM.02034-17 (PMC5835749; doi:10.1128/AEM.02034-17)
Supplement: Supplemental material [file supp_84_6_e02034-17__index.html]

Supplemental material 

# Lipidomics of Thalassiosira pseudonana under Phosphorus Stress Reveal Underlying Phospholipid Substitution Dynamics and Novel Diglycosylceramide Substitutes

## Supplemental material

- Supplemental file 1 -

  Macronutrient concentrations in the cultures through time (Fig. S1); total glycerophosphatidylcholine, total glycerophosphatidylglycerol), total glycerophosphatidylethanolamine, total diacylglycerol, and total sulfoquinovosyldiacylglycerol per cell under P+ and P− conditions with the progression of time (Fig. S2); heatmap of correlation coefficients from pairwise comparisons between different lipid classes at each time point and each treatment as a measure of fatty acid compositional similarity (Fig. S3); pairwise regression analysis of PC degradation and DGCC synthesis, per milliliter culture volume, between subsequent time points (Fig. S4); chemical structure assignment of (Gly)2Cer(d18:3/24:0) and supporting MS2 fragmentation data in negative and positive ion modes (Fig. S5); untargeted screen of the *T. pseudonana* lipidome subjected to P stress (Fig. S6).

  PDF, 1.1M
