# Supplementary material for: Lipidomics of Thalassiosira pseudonana under Phosphorus Stress Reveal Underlying Phospholipid Substitution Dynamics and Novel Diglycosylceramide Substitutes
Source: Appl Environ Microbiol. 2018 Mar 1;84(6):e02034-17. doi: 10.1128/AEM.02034-17 (PMC5835749; doi:10.1128/AEM.02034-17)
Supplement: Supplemental material [file AEM.02034-17_zam006188368s1.pdf]

Lipidomics of *Thalassiosira pseudonana* Under Phosphorus Stress Reveal Underlying Phospholipid Substitution Dynamics and Novel Diglycosylceramide Substitutes

SUPPLEMENTARY MATERIALS

Running Title: P Stressed Diatom Lipids

Jonathan E. Hunter[1,2†], Joost Brandsma[3], Marcus K. Dymond[4], Grielof Koster[3], C. Mark Moore[1], Anthony D. Postle[3], Rachel A. Mills[1] and George S. Attard[5].

1. Ocean and Earth Science, University of Southampton, National Oceanography Centre Southampton, European Way, Southampton, SO14 3ZH, United Kingdom
2. Institute for Life Sciences, University of Southampton, SO17 1BJ, United Kingdom
3. Faculty of Medicine, University of Southampton, Southampton General Hospital, Tremona Road, Southampton, SO16 6YD, United Kingdom
4. Division of Chemistry, School of Pharmacy and Biomolecular Sciences, University of Brighton, Brighton, BN2 4GJ, United Kingdom
5. Chemistry, University of Southampton, Southampton, SO17 1BJ, United Kingdom

†Corresponding Author: jhunter@whoi.edu; Current Address: Woods Hole Oceanographic Institution, Woods Hole, MA 02543-1050, United States of America

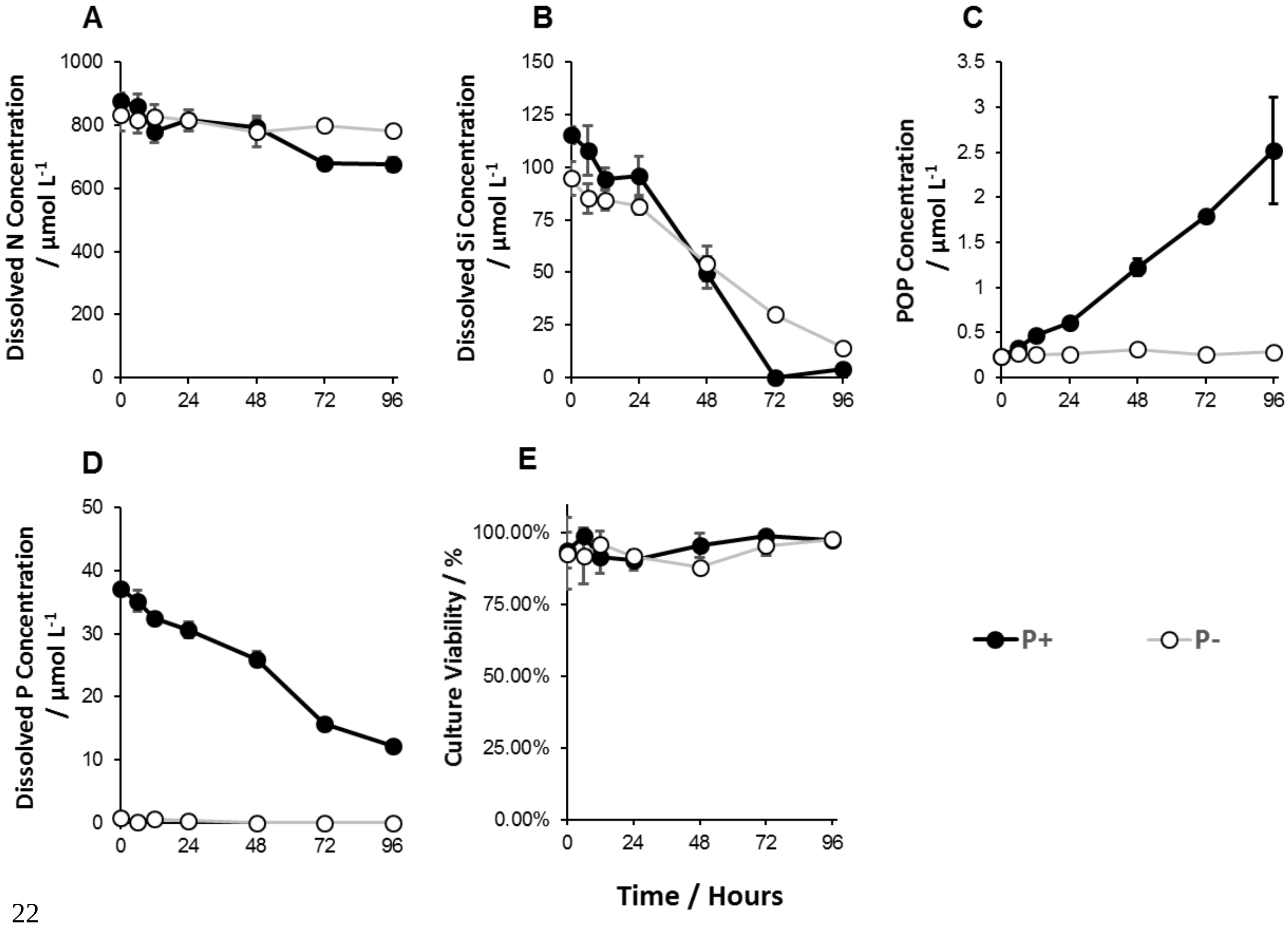

Supplementary Figure 1 - Macronutrient concentrations in the cultures through time. Dissolved phosphorus concentration in the growth media (A); dissolved silicon concentration in the growth media (B); particulate organic phosphorus concentration per litre growth media (C); dissolved phosphorus concentration in the growth media (D) and percentage viability of the T. pseudonana cells in culture (E). Data include P+ and P- conditions, with the progression of time. Data are the mean of n = 3 biological replicates, with error bars of 1 standard deviation.

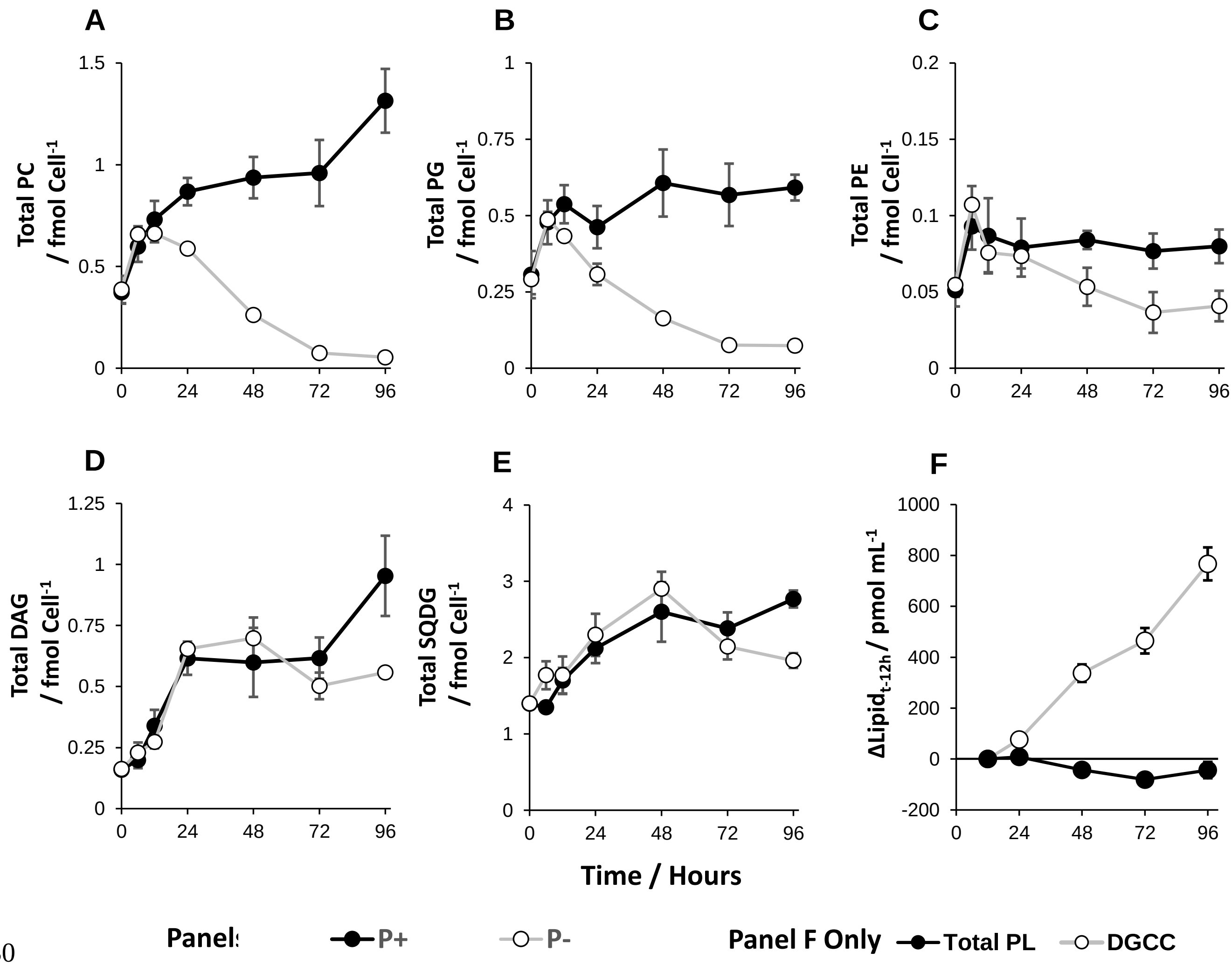

Supplementary Figure 2 – Total glycerophosphatidylcholine (PC, A), total glycerophosphatidylglycerol (PG, B), total glycerophosphatidylethanolamine (PE, C), total diacylglycerol (DAG, D) and total sulfoquinovosyldiacylglycerol (SQDG, E) per cell under P+ and P- conditions, with the progression of time. Panel (F) depicts the change in total lipid quantity (total phospholipid (P-Lipid) and DGCC) per mL culture ($Lipid_{t-12h}$*), between time t and 12 h, in the P- cultures (A). y < 0 indicates a net degradation and loss of the total phospholipids, y = 0 indicates a constant quantity (no net synthesis or degradation), y > 0 indicates biosynthesis and net increase in total lipid quantity. Values are relative to 12 h, observed as the initiation of P stress and consequently DGCC biosynthesis and P-Lipid*

substitution/degradation in the P- cultures. Data are the mean of n = 3 biological replicates, with error bars of 1 standard deviation.

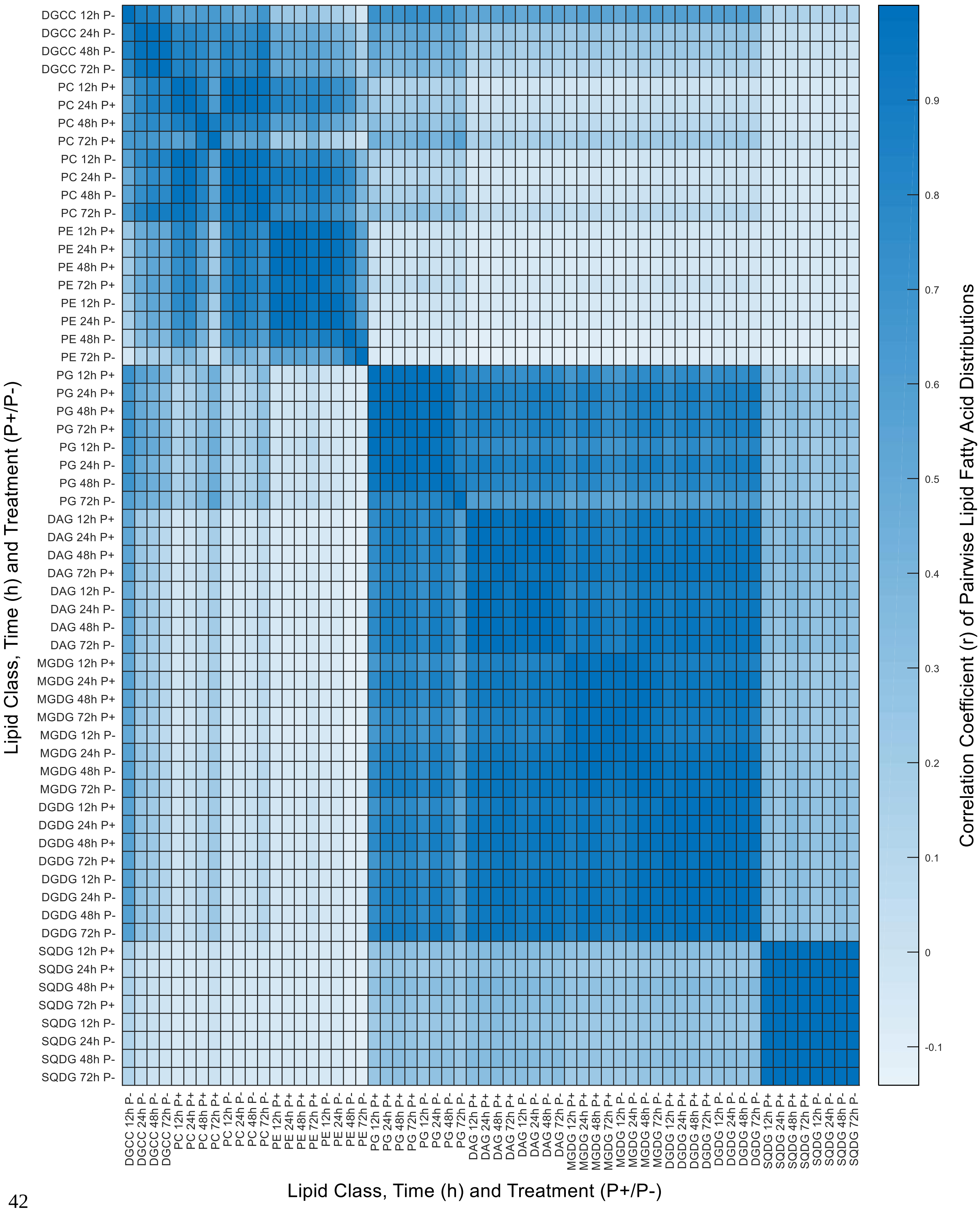
DGCC 12h P-
DGCC 24h P-
DGCC 48h P-
DGCC 72h P-
PC 12h P+
PC 24h P+
PC 48h P+
PC 72h P+
PC 12h P-
PC 24h P-
PC 48h P-
PC 72h P-
PE 12h P+
PE 24h P+
PE 48h P+
PE 72h P+
PE 12h P-
PE 24h P-
PE 48h P-
PE 72h P-
PG 12h P+
PG 24h P+
PG 48h P+
PG 72h P+
PG 12h P-
PG 24h P-
PG 48h P-
PG 72h P-
DAG 12h P+
DAG 24h P+
DAG 48h P+
DAG 72h P+
DAG 12h P-
DAG 24h P-
DAG 48h P-
DAG 72h P-
MGDG 12h P+
MGDG 24h P+
MGDG 48h P+
MGDG 72h P+
MGDG 12h P-
MGDG 24h P-
MGDG 48h P-
MGDG 72h P-
DGDG 12h P+
DGDG 24h P+
DGDG 48h P+
DGDG 72h P+
DGDG 12h P-
DGDG 24h P-
DGDG 48h P-
DGDG 72h P-
SQDG 12h P+
SQDG 24h P+
SQDG 48h P+
SQDG 72h P+
SQDG 12h P-
SQDG 24h P-
SQDG 48h P-
SQDG 72h P-
Lipid Class, Time (h) and Treatment (P+/P-)
Lipid Class, Time (h) and Treatment (P+/P-)
Correlation Coefficient (r) of Pairwise Lipid Fatty Acid Distributions
0.9
0.8
0.7
0.6
0.5
0.4
0.3
0.2
0.1
0
-0.1

Supplementary Figure 3: Heatmap of correlation coefficients (r) from pairwise comparisons between different lipid classes at each time point (between 12 and 72 h) and each treatment (P+ and P-), as a measure of fatty acid compositional similarity. The correlation coefficients were calculated from percentage relative abundances of each individual lipid species. As the % relative abundances sum to 100 in both cases, the fatty acyl similarity of a pair can be simply assessed upon r. Data are the mean of n = 3 biological replicates.

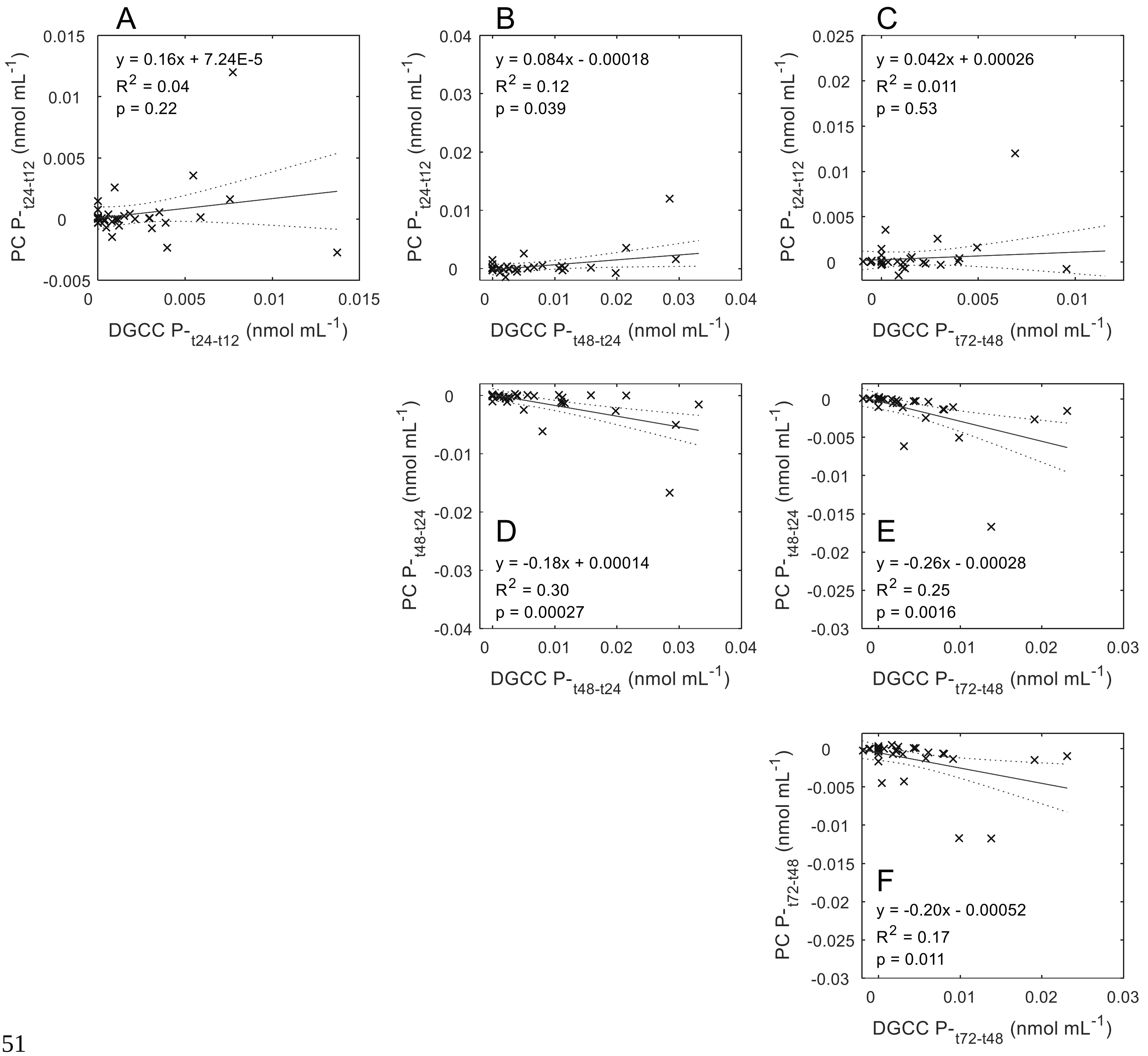

Supplementary Figure 4 – Pairwise regression analysis of PC degradation and DGCC synthesis, per mL culture volume, between subsequent time points from 12 to 72 h. Data points are individual lipid species, of the same fatty acid composition, between the two lipid classes. This analysis was performed to look for evidence of the recycling of diacylglyceride substructures, liberated by the degradative breakdown of PC and funnelled into the synthesis of the substitute lipid DGCC. Such a relationship would be evident here as a significant anticorrelation, as observed in the DGCC P- $_{t48\text{-}t24}$*/PC P-* $_{t48\text{-}t24}$*; DGCC P-* $_{t72\text{-}t48}$*/PC P-* $_{t48\text{-}24}$*; and DGCC P-* $_{t72\text{-}t48}$*/PC P-* $_{t72\text{-}t48}$ *cases (panels D, E, and F). Data are the mean of n = 3*

biological replicates. The dashed lines represent the 95% confidence bounds of the linear regression model.

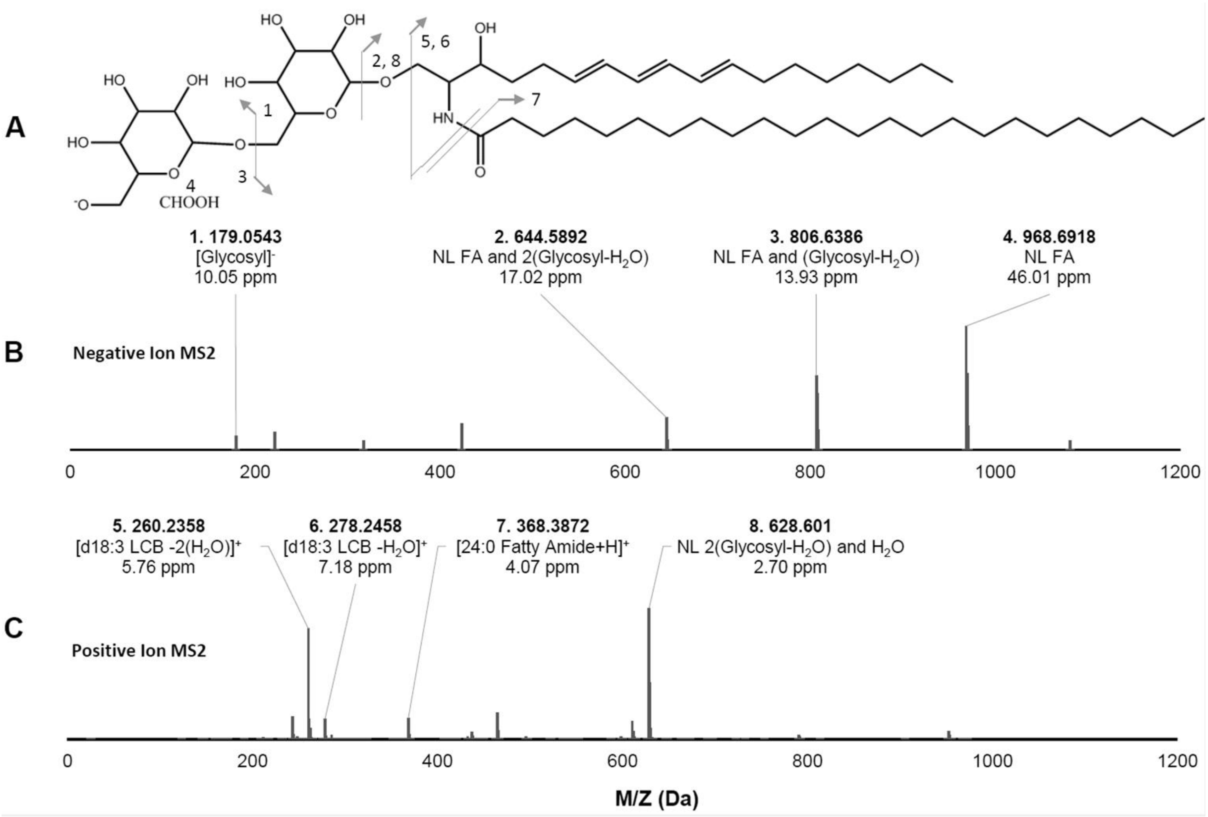

Supplementary Figure 5 - Chemical structure assignment of (Gly)$_2$Cer(d18:3/24:0) (A) and supporting MS2 fragmentation data in negative (B) and positive (C) ion mode. Spectra from a single representative P- sample. Hydroxyl group and unsaturation regio- and stereochemistry was not resolved. Arrows indicate fragmentation and the direction of the charged ion position. The negative ion MS2 (B) revealed fragment ions of 968.6918, 806.6386, 644.5892 and 179.0543 Da, corresponding to a neutral loss of the formic acid (CHOOH) adduct, neutral loss of CHOOH and a glycosyl-H2O, neutral loss of CHOOH and two glycosyl-H2O units and a glycosyl fragment respectively. Knowledge of the CHOOH adduct and the retention time was used to identify the [M+H]+ equivalent in positive ion mode and its respective MS2 fragmentation spectrum. In positive ion mode (C), MS2 fragment ions of 628.6010, 368.3872, 278.2458 and 260.2358 corresponded to a neutral loss

of two glycosyl moieties, a 24:0 fatty amide fragment ion and a d18:3 long chain base minus one and two H2O respectively.

(A)
Positive

Database Assignment and Corroborating MS2 Evidence:

**MZ (Da)**	**R.T.**	**P Val.**	**P+**	**P-**	**Adduct**	**Assignment**	**PPM Diff.**	**MS2 Fragments**
774.5897	11.5	1.04E-05	0.00	1.00	$(M+H)^+$	DGCC(20:5/16:0)	2.39	D184, 20:5 and 16:0 NL Fatty Ketene
536.3573	5.0	2.19E-03	0.00	1.00	$(M+H)^+$	LDGCC(20:5)	-1.57	D104, 20:5 NL Fatty Ketene
800.6057	11.9	5.98E-05	0.00	1.00	$(M+H)^+$	DGCC(22:6/16:0)	2.71	D104, 22:6 and 16:0 NL Fatty Ketene
562.3729	5.4	8.74E-03	0.00	1.00	$(M+H)^+$	LDGCC(22:6)	-1.65	D104, 22:6 NL Fatty Ketene
820.5735	10.6	1.02E-04	0.00	1.00	$(M+H)^+$	DGCC(20:5/20:5)	1.59	D104, 20:5 NL Fatty Ketene
846.5897	11.0	1.48E-04	0.00	1.00	$(M+H)^+$	DGCC(22:6/20:5)	2.20	D104, 22:6 NL Fatty Ketene
772.5719	10.8	2.91E-03	0.00	1.00	$(M+H)^+$	DGCC(20:5/16:1)	-0.32	D104, 20:5 NL Fatty Ketene
748.5718	11.0	1.81E-03	0.00	1.00	$(M+H)^+$	DGCC(18:4/16:0)	-0.50	D104, 18:4 and 16:0 NL Fatty Ketene
726.5891	11.7	8.79E-04	0.00	1.00	$(M+H)^+$	DGCC(16:1/16:0)	1.68	D104, 16:1 and 16:0 NL Fatty Ketene
746.5589	10.5	2.22E-03	0.00	1.00	$(M+H)^+$	DGCC(20:5/14:0)	3.10	D104, 20:5 and 14:0 NL Fatty Ketene
510.3410	4.8	9.59E-03	0.00	1.00	$(M+H)^+$	LDGCC(18:4)	-3.04	D104, 18:4 NL Fatty Ketene
724.5720	11.1	4.12E-03	0.00	1.00	$(M+H)^+$	DGCC(16:1/16:1)	-0.20	D104, 16:1 NL Fatty Ketene
796.7424	20.7	7.97E-04	0.00	1.00	$(M+NH_4)^+$	TAG(16:0/16:0/14:0)	4.41	16:0 and 14:0 NL FA+NH3
798.5871	11.1	1.03E-03	0.00	1.00	$(M+H)^+$	DGCC(20:5/18:2)	-0.96	D104, 20:5 and 18:2 NL Fatty Ketene
794.5575	10.1	2.88E-02	0.00	1.00	$(M+H)^+$	DGCC(20:5/18:4)	1.23	D104, 20:5 NL Fatty Ketene

(B)
Negative

Database Assignment and Corroborating MS2 Evidence:

**MZ (Da)**	**R.T.**	**P Val.**	**P+**	**P-**	**Adduct**	**Assignment**	**PPM Diff.**	**MS2 Fragments**
818.5771	11.5	4.41E-04	0.00	1.00	$(M+HAc-CH_3)^-$	DGCC(20:5/16:0)	-2.18	20:5 and 16:0 FA Daughter Fragments
844.5949	11.9	7.12E-04	0.00	1.00	$(M+HAc-CH_3)^-$	DGCC(22:6/16:0)	0.49	22:6 and 16:0 FA Daughter Fragments
890.5801	11.1	1.77E-04	0.00	1.00	$(M+HAc-CH_3)^-$	DGCC(22:6/20:5)	1.42	20:5 FA Daughter Fragment
770.5778	11.6	1.87E-03	0.00	1.00	$(M+HAc-CH_3)^-$	DGCC(16:1/16:0)	-1.39	16:1 and 16:0 FA Daughter Fragments
816.5621	10.8	2.20E-05	0.00	1.00	$(M+HAc-CH_3)^-$	DGCC(20:5/16:1)	-1.35	20:5 and 16:1 FA Daughter Fragments
792.5624	11.1	4.49E-04	0.00	1.00	$(M+HAc-CH_3)^-$	DGCC(18:4/16:0)	-1.02	18:4 and 16:0 FA Daughter Fragments
1174.7555	12.9	1.30E-05	0.00	1.00		Unknown		
1014.7112	13.6	6.17E-03	0.00	1.00	$(M+FA-H)^-$	$(Gly)_2$Cer(d18:3/24:0)	1.34	See Figure 3
532.3505	5.1	4.67E-02	0.00	1.00	$(M+HAc-CH_3)^-$	LDGCC(16:1)	2.49	16:1 FA Daughter Fragment
796.5931	11.9	3.46E-04	0.00	1.00	$(M+HAc-CH_3)^-$	DGCC(18:2/16:0)	-1.72	18:2 and 16:0 FA Daughter Fragments
595.5395	12.7	4.14E-03	0.00	1.00		Unknown		
838.5529	10.1	4.59E-03	0.00	1.00	$(M+HAc-CH_3)^-$	DGCC(20:5/18:4)	6.38	Coelution with Identified +ve Ion
623.5695	13.6	8.15E-03	0.14	0.86		Unknown		
819.5296	11.3	3.72E-02	0.22	0.78	$(M-H)^-$	SQDG(34:1)	-0.26	DB Match and Diagnostic R.T. Only
694.6305	15.4	1.28E-02	0.27	0.73		Unknown		

Supplementary Figure 6 - Untargeted screen of the T. pseudonana lipidome subject to P stress. Detected ions were ranked based upon normalised differential abundance

(Quantity(P-)/(Quantity(P+) + Quantity(P-)) therefore those at the top are most strongly increased subject to P stress. Panel A displays positive ions and Panel B displays negative ions. R.T. represents chromatographic retention time, P value was determined by unpaired, two sample equal variance T-test. Assignment represents the lipid identity, PPM Diff. the difference between the observed and predicted M/Z, and MS^2 fragments outlines the observed fragments under $AutoMS^2$ in support of the designated assignment. L- refers to a lyso-species (bearing 1 rather than 2 fatty acids), NL = neutral loss, HAc = Acetic Acid and FA = Formic Acid. Data represent the mean of biological triplicate samples. Assignments represent the primary fatty acyl configuration, as determined by the abundance of the fatty acyl fragments in the MS^2 spectra.
